# Supplementary figures and images for: Profiles of stress hormones in relation to DENV serotypes among dengue-positive patients
Source: PLoS One. 2026 Apr 24;21(4):e0344532. doi: 10.1371/journal.pone.0344532 (PMC13108801; doi:10.1371/journal.pone.0344532)

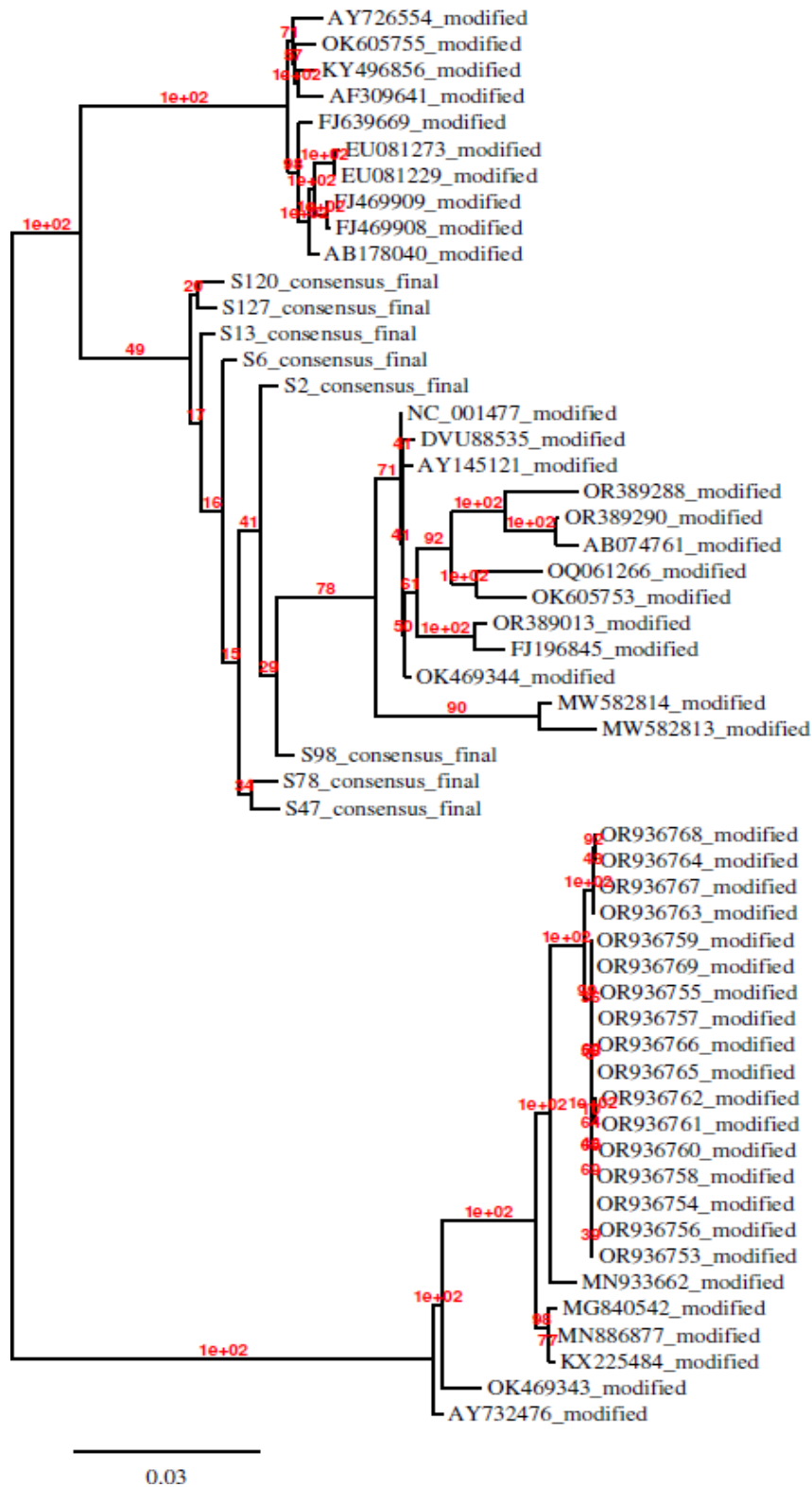

**S1 Figure: Maximum likelihood tree showing phylogenetic relationships among DENVV-1 samples.**

Supplement: S1 Fig — (PDF) [file pone.0344532.s001.pdf]
