# Supplementary material for: Profiles of stress hormones in relation to DENV serotypes among dengue-positive patients
Source: PLoS One. 2026 Apr 24;21(4):e0344532. doi: 10.1371/journal.pone.0344532 (PMC13108801; doi:10.1371/journal.pone.0344532)
Supplement: Attachment — Submitted filename: Response to Reviewers.docx [file pone.0344532.s005.docx]

**Response Sheet**

We would like to sincerely thank the reviewers for their time, effort, and valuable comments in reviewing our work. Their constructive feedback has greatly helped us refine and improve the manuscript, making it more meaningful and useful for the scientific community. Please find below our detailed responses to the reviewers’ comments, addressed point by point.

**Reviewer No 02:**

1. The title suggests a mechanistic or causal relationship ("association") but the study design does not support causality - this is a cross-sectional observational study. Please adjust the title to reflect the descriptive nature of the analysis, e.g., "Profiles of Stress Hormones in Relation to DENV Serotypes among Dengue-Positive Patients."

Response: *Title, Page no. 2, Line 02*

In accordance with the reviewer’s suggestion, we have revised the title to “**Profiles of Stress Hormones in Relation to DENV Serotypes among Dengue-Positive Patients**.”

2. The abstract promises a novel exploration of hormonal response to DENV genotypes, but the results do not show statistically meaningful differences. Furthermore, it claims "modest positive correlation" but does not substantiate it with statistical significance or effect sizes. Please revise the abstract to reflect the exploratory and non-significant nature of the findings, and include correlation coefficients with p-values or confidence intervals where possible.

Response: *Abstract, Page no. 2, Line no. 47-52 & 56-58*

We have revised the abstract to better reflect the exploratory and descriptive nature of the study. Specifically, we have now included correlation coefficients with p-values to substantiate the reported associations. “Notably, comparisons across different DENV serotypes did not reveal statistically significant differences in stress hormone response, indicating only descriptive trends. Spearman’s correlation analysis showed weak, non-significant associations between viral load and cortisol (r = 0.184, p = 0.170) and between viral load and epinephrine (r = 0.165, p = 0.224). Statistically significant but modest negative correlation was observed only between viral load and hemoglobin (r = -0.228, p = 0.005).” “However, as most associations were exploratory and not statistically significant. The descriptive trends identified here highlight the need for larger, longitudinal studies to better define the relationship between stress hormones, viral load, and dengue severity.” These values have been incorporated into the revised abstract and respective results section to provide full transparency regarding the non-significant nature of most associations.

3. Large portions of the epidemiological background in the Introduction are devoted to general dengue epidemiology, which adds little to the justification of the study. Please streamline the background and focus instead on highlighting gaps in the literature related to host hormonal responses by serotype or genotype.

Response: *Introduction Section, Page no. 04, Line 104-116*

In the revised manuscript, we have streamlined the epidemiological background to a concise summary and shifted the focus to highlighting gaps in the literature regarding host hormonal responses. “Although dengue has been widely studied in terms of epidemiology and viral diversity, little attention has been given to host endocrine responses. For example, cortisol has been linked to disease severity, with lower levels reported in children with severe dengue shock syndrome [10, 14], but such studies did not assess differences by serotype or genotype. To date, no research has systematically evaluated stress hormone responses (cortisol and adrenaline) across DENV serotypes. Addressing this gap, our study investigates stress hormone profiles in relation to DENV serotype-genotype characterization in dengue-positive patients from Pakistan.

We have previously reported the whole-genome phylogenetic characterization of DENV-1 strains circulating in Pakistan [14]. Building on that foundation, the present study takes a more integrative approach. Specifically, we investigate host endocrine responses (cortisol and adrenaline) in dengue-positive patients and compare these with viral load, serotype distribution, and hematological indices.”

To our knowledge, no study has systematically evaluated stress hormone dynamics (cortisol and adrenaline) across different DENV serotypes/genotypes. We have now emphasized this gap in the revised Introduction to better justify the rationale of our study.

4. The rationale behind linking stress hormones (cortisol, adrenaline) with specific DENV genotypes is not grounded in any referenced theoretical or biological framework. There is no citation suggesting that serotype-specific viral behavior would mechanistically affect the HPA axis. Thus, authors must provide a mechanistic hypothesis or at least cite prior work showing links between DENV variants and neuroendocrine responses to strengthen their premise.

Response: *Introduction Section, Page no. 3-4, Line 93-100*

We have now added new references to the Introduction to strengthen the scientific premise. “This interaction between viral infection, cytokine signaling, and HPA axis activation is well documented [9]. In dengue specifically, altered cortisol levels have been reported in relation to disease severity [10, 11], although prior studies have not examined variation across serotypes or genotypes. Elevated pro-inflammatory cytokines such as TNF-α, IL-6, and IL-17, together with differential IL-10 responses in dengue hemorrhagic versus classical dengue, have been shown to play a major role in severity progression. These findings underscore the immune axis in dengue pathogenesis and provide theoretical support for exploring potential interactions between stress hormones and cytokine responses [12].”

5. Previous studies on hormonal response in infectious diseases exist, but this work neither builds on nor properly distinguishes itself from them. So what differentiates this study must be clearly articulated whether it is geographic specificity, inclusion of whole-genome phylogenetics, or a novel analytic approach.

Response: *Introduction Section, Page no. 04, line 104-116*

We have revised the manuscript to explicitly highlight how the current study differs from both earlier work on hormonal responses in infectious diseases and from our own previously published genomic study. “Although dengue has been widely studied in terms of epidemiology and viral diversity, little attention has been given to host endocrine responses. For example, cortisol has been linked to disease severity, with lower levels reported in children with severe dengue shock syndrome [10-14], but such studies did not assess differences by serotype or genotype. To date, no research has systematically evaluated stress hormone responses (cortisol and adrenaline) across DENV serotypes. Addressing this gap, our study investigates stress hormone profiles in relation to DENV serotype-genotype characterization in dengue-positive patients from Pakistan.

We have previously reported the whole-genome phylogenetic characterization of DENV-1 strains circulating in Pakistan [14]. Building on that foundation, the present study takes a more integrative approach. Specifically, we investigate host endocrine responses (cortisol and adrenaline) in dengue-positive patients and compare these with viral load, serotype distribution, and hematological indices.”

*(Discussion Section, Page no. 11, line 355-360)*

“Unlike our previous study, which focused solely on genomic characterization and phylogenetic diversity of DENV-1 strains in Pakistan [14], this work integrates quantification of stress hormones (cortisol and adrenaline) and hematological parameters, correlating them with serotype and viral load. This virologic-endocrine approach provides novel insights into host response and highlights the interplay between viral diversity and host hormonal dynamics, a framework not previously reported in Pakistan.”

6. This is a descriptive, cross-sectional design, which is not sufficient to make any inference on interaction or impact between hormones and genotypes. Thus, conclusions implying serotype-hormone effects should be reworded to emphasize association without assuming directionality or causality.

Response: *Results, discussion and conclusion sections*

We have carefully revised the manuscript to avoid wording that could imply causality or directionality, in the following sections.

*Results (line no. 266 - 267)*

“Only the associations between viral load, and hemoglobin was found statistically significant (p < 0.05).”

*Discussion (line no. 345 - 348)*

“In our investigation, we discovered a correlation between the measured blood parameters and stress hormones and virus load. Hemoglobin levels showed a modest but statistically significant inverse relationship with viral load.”

*Conclusion sections (line no. 382-387)*

“In wrapping up our study, we observed that patients infected with DENV-2 had the lowest platelet counts, suggesting this serotype may be associated with greater hematological impact in our cohort.

While modest correlations were observed between blood parameters, stress hormones, and viral load, the cross-sectional design limits conclusions regarding their impact on illness severity.”

7 There's no sample size justification or power calculation, making it unclear whether the study is adequately powered to detect the hypothesized associations. Please include a power analysis to justify whether the study was sufficiently powered to detect differences in hormone levels between serotypes.

Response: *Discussion section, Page no. 12, line 361 - 367*

A formal power calculation was not performed at the study’s inception, as this was an exploratory study conducted during a dengue outbreak. Instead, the study was designed to include all dengue-positive patients presenting to Dow University Hospital within the defined study period (September–December 2022), resulting in a total of 150 cases and 15 healthy controls. We recognize that the absence of a prior sample size justification limits the ability to claim adequate statistical power for detecting associations between stress hormone levels and DENV Serotypes.

To address this, we added the following:

“Our study has certain limitations. First, the control group size was relatively small (n = 15) compared to the patient group (n = 150). Controls were recruited from healthy individuals during the outbreak period, but resource constraints limited their number. As a result, the study may have been underpowered to detect smaller associations, although it provided adequate data for descriptive analyses. Therefore, the findings should be interpreted as exploratory. Future larger, multi-center studies with formal sample size calculations will be needed to confirm and extend these preliminary observations.”

8. The study was done in a limited geographic region and over a short time period. External validity is not addressed. This could limit generalizability to other settings or populations. Please clarify this limitation in the discussion section.

Response: *Discussion Section, Page no. 12, Line no. 371 - 376*

“Furthermore, the study was conducted during a peak outbreak session attending the government regulated tertiary care hospital. Though this may limit the external validity and generalizability of our findings to other populations or epidemiological contexts, Dow University Hospital serves to cater multi-ethnic patients from wider localities. Although these findings cannot be generalized to a global population, they do not appear to be confined to any particular sector or subgroup.”

9. There is an ambiguity in patient selection and controls. The description lacks clarity on how negative controls were defined and matched. Thus, authors must clarify inclusion/exclusion criteria for controls, matching strategy (if any), and whether potential confounders were balanced between groups.

Response: Materials and Methods, Page no. 5, line 129 - 135

We recognize the limitations and have clarified the selection criteria for cases and controls in the revised manuscript as “During the outbreak, dengue-positive cases were recruited following clinical suspicion by physicians and confirmed through NS1 antigen and RT-PCR testing. Negative controls were recruited from individuals testing negative for dengue in the same setting, using random selection according to resource availability. Detailed fever history was not systematically available, which we recognize as a limitation. Although one-to-one matching was not possible, group-level comparability in age and sex distribution was maintained, and baseline hematological indices were reviewed to minimize imbalances”.

10 The use of correlational analysis with vague effect sizes and no correction for multiple testing is scientifically weak. For example, reporting weak positive correlations without FDR or Bonferroni correction inflates type I error. Authors may fix this by applying correction methods and reporting adjusted p-values.

*Materials and Methods, Page no. 7, line no. 223-227*

*Table no 6, Page no, 27*

*Figure 3, Page no, 18*

*Figure 4 Page no, 19*

Response:

As suggested, Bonferroni correction for multiple comparisons was applied to all correlational analyses, and Bonferroni-adjusted p-values are now reported. This has been added to the Statistical Analysis section, and the relevant table (Table 6) has also been updated. p<0.05 was considered statistically significant in all analysis such as Figure 3 & 4; Table 3 – 6.

11. The discussion implies biological importance of observed hormone differences without theoretical or empirical support. This could mislead readers into overinterpreting minor variations. Please tone down speculative interpretations unless supported by literature or strong statistical evidence.

Response:

As noted in our response to Comment 06, we have carefully revised the Results, Discussion, and Conclusion sections to remove causal or mechanistic language and to emphasize that our findings are exploratory associations rather than definitive biological effects. In line with this revision, speculative interpretations of hormone differences have been toned down, and the text now clearly frames these observations as preliminary and hypothesis-generating, requiring further validation in larger studies.

12. Please improve the comparison with prior literature. Particularly, the manuscript does not engage with earlier studies on hormonal changes in dengue, missing an opportunity to contextualize findings in light of immune-endocrine interactions in viral infections.

Response:

As noted in our response to Comment 04, we have revised the Introduction to incorporate references on immune–endocrine interactions in viral infections and earlier studies reporting cortisol dynamics in dengue patients. In addition, we have expanded the Discussion to compare our findings with these prior reports, while carefully framing our observations as exploratory associations (as also addressed under Comment 06 and Comment 11). Together, these revisions strengthen the contextualization of our study and clarify how our work contributes to the existing body of knowledge by addressing a gap not previously explored the association of stress hormone profiles with DENV serotypes/genotypes.

13. I think part of the discussion is speculative in interpretation. The phylogenetic inference about multiple introductions of DENV into Pakistan does not align with the main hypothesis and seems tangential. To address this, authors may either connect the phylogenetic insights to disease severity or patient outcomes or move this section to supplementary results if not central to the main research question.

Response: *Page no 21*

The phylogenetic inference, while informative, is not central to our primary hypothesis regarding stress hormone profiles. In response, we have moved the phylogenetic tree to the Supplementary Materials and streamlined the related discussion to a brief contextual note rather than a central interpretation. This ensures that the main text remains focused on the study’s core objectives, while still allowing readers access to the phylogenetic analysis for completeness. **Supplementary Figure S1: Maximum likelihood tree showing phylogenetic relationships among DENVV-1 samples.**

14. Authors claim about potential application in outbreak management. Is this accurate? Given the study design is observational in nature, and most of the findings are hard to implicate to somewhat actionable, this claim should be reworded to suggest that findings are hypothesis-generating rather than directly informing public health strategies.

Response: *Conclusion. Page no. 12, line 392-393.*

As per the respective suggestion, we have revised the relevant statements in the Conclusion section to clarify that our results are hypothesis generating and exploratory in nature, offering preliminary insights that may guide future research rather than actionable public health recommendations. This revision aligns with the changes already made in response to Comments 06 and 11, where we reframed our interpretations to emphasize association and exploratory trends rather than causality or application. Additionally we have also tone down the last sentence in the concluding “These findings offer preliminary insights into the interplay of dengue serotypes and may serve as a basis for generating hypotheses about their implications for infection outcomes.”

15. Why do you think, or not, the study can warrant changes in diagnostic, clinical, or public health approaches? The current data are not robust or generalizable enough to warrant such changes. However, it may contribute to the body of exploratory biomarker research pending validation in larger, multi-site cohorts.

Response: *Discussion section, Page no. 11-12, line 361 - 379*

In line with the reviewer’s suggestion, we have revised the Discussion and Conclusion

“Our study has certain limitations. First, the control group size was relatively small (n = 15) compared to the patient group (n = 150). Controls were recruited from healthy individuals during the outbreak period, but resource constraints limited their number. As a result, the study may have been underpowered to detect smaller associations, although it provided adequate data for descriptive analyses. Therefore, the findings should be interpreted as exploratory. Future larger, multi-center studies with formal sample size calculations will be needed to confirm and extend these preliminary observations. Second, detailed phase-specific fever data were unavailable, which restricted our ability to evaluate fluctuations in stress hormone levels across different stages of illness. While demographics, hematological indices, study location, and study period were reported, unmeasured confounders such as timing of sample collection, comorbidities, and concurrent medications may have influenced cortisol and adrenaline levels. Furthermore, the study was conducted during a peak outbreak session attending the government regulated tertiary care hospital. Though this may limit the external validity and generalizability of our findings to other populations or epidemiological contexts, Dow University Hospital serves to cater multi-ethnic patients from wider localities. Although these findings cannot be generalized to a global population, they do not appear to be confined to any particular sector or subgroup. Taken together, these limitations underscore the exploratory nature of our work. Nevertheless, examining the interactions between stress hormones and dengue serotypes may provide insights that may contribute to body of exploratory biomarker research.”

16. I worry about the use of only non-parametric tests limits analytical depth, and there's no multivariate control for confounders. Authors should consider multivariable regression models adjusting for age, sex, infection status, and time since symptom onset to better elucidate relationships.

Response: *Materials and Methods Section, Page no., Line no. 222-226*

We agree that multivariate regression models are important tools for controlling confounders. In our study, we used non-parametric tests, including Spearman correlation, as these were most appropriate given the data distribution, the relatively small control group, and the study objectives. In revised manuscript updated, as “In addition, when a statistical association was found, pairwise analysis was performed using Dunn’s post hoc test with Bonferroni adjustment. Spearman’s correlation was used to assess the associations between viral load, stress markers, and hematological parameters in dengue-infected patients, and Bonferroni-adjusted p-values are reported.” Also shown in (Table 4 & 6).

17. More details should be added to patient demographics potentially influencing cortisol/adrenaline levels (e.g., time of sample collection, medications, comorbidities). These factors could significantly confound hormone measurements and must be accounted for or discussed as limitations.

Response: *Discussion Section, Page no. 12, Line no. 369-371*

Patient demographics including age and sex, hematological indices, and the geographic setting (Karachi, Pakistan) are provided in the revised manuscript. In addition, the study period and time span of sample collection have already been described in the Methods section. However, we acknowledge that some potentially relevant factors such as exact time of day of sample collection, comorbidities, and concurrent medication use were not systematically recorded, which could have influenced cortisol and adrenaline levels. We have now added this point explicitly in the Discussion under the limitations section as “While demographics, hematological indices, study location, and study period were reported, unmeasured confounders such as timing of sample collection, comorbidities, and concurrent medications may have influenced cortisol and adrenaline levels.” Also shown in (Table 1).

18. Consider to add more relevant studies in the discussion, such as by stating: "Although our cohort is based in Karachi rather than Mexico, global shifts in serotype prevalence—as documented by Mendoza-Cano et al. (2025)—underscore the relevance of investigating whether new serotypes (e.g., DENV-3) elicit different host physiological responses.” Ref: PLOS One. 2025 May 22;20(5):e0324754

Response: *Discussion section, Page no. 10, line 307 - 310*

We have cited/incorporated the recommended studies as “Although our cohort is based in Karachi rather than Mexico, global shifts in serotype prevalence as documented by Mendoza-Cano et al. (2025) underscore the relevance of investigating whether newly emerging serotypes (e.g., DENV-3) may elicit distinct host physiological and hormonal responses [28].”

19. Introduction can benefit from the addition of: “As example in Indonesia, dengue control efforts are increasingly compromised by widespread resistance of Aedes aegypti to standard insecticides, notably pyrethroids and organophosphates—especially across Java and neglected rural areas—highlighting the need for sustainable alternatives such as plant-based biolarvicides (Kasman et al., 2025) [e-1819]”

Response: *Introduction section, Page no. 3, line 86 - 89*

We have cited/incorporated the recommended studies as “In Indonesia, dengue control efforts are increasingly compromised by widespread resistance of Aedes aegypti to standard insecticides, notably pyrethroids and organophosphates, particularly across Java and neglected rural areas. This highlights the urgent need for sustainable alternatives such as plant-based biolarvicides [8].”

20. To lay the biological foundation for exploring hormonal vs. immune biomarker patterns, authors may incorporate the following: “Elevated pro-inflammatory cytokines such as TNF-α, IL-6, and IL-17, along with differential IL-10 responses in dengue hemorrhagic versus classical dengue, highlight the immune axis in severity progression—supporting theoretical rationale for exploring stress hormone–cytokine interplay (Masyeni et al., Narra J 2024;4(1):e309)”

Response: *Introduction section, Page no. 3 & 4, line 96 - 100*

We have now incorporated the study by Masyeni et al. (2024) into the Introduction as “Elevated pro-inflammatory cytokines such as TNF-α, IL-6, and IL-17, together with differential IL-10 responses in dengue hemorrhagic versus classical dengue, have been shown to play a major role in severity progression. These findings underscore the immune axis in dengue pathogenesis and provide theoretical support for exploring potential interactions between stress hormones and cytokine responses [12]”

21. Authors have not made the underlying data publicly available as recommended by PLOS ONE. This contradicts the journal’s data availability policy, which emphasizes transparency and reproducibility. Authors must deposit the complete dataset in a public repository or provide access upon request, along with metadata and a clear data availability statement in the manuscript.

Response:

We have now addressed this by updating the manuscript to include a Data Availability Statement in line with the PLOS ONE policy. The statement specifies that the de-identified dataset underlying this study is [publicly available in a repository / available upon reasonable request from the corresponding author], thereby ensuring transparency and reproducibility.

**Reviewer # 3:**

-Address Unequal Group Sizes:

The unbalanced distribution of serotypes (DENV-1: 80%; DENV-2: 16%; DENV-3: 0.7%; co-infections: 3.3%) may limit statistical power for comparisons involving DENV-3 and co-infections. Implement post-hoc tests (e.g., Dunn’s test) following the Kruskal-Wallis test to identify specific differences between serotypes, accounting for unequal group sizes.

Response: *Materials and Methods Section, Page no. 4, line 219-220, 224 – 228*

Thank you for your comment. As suggested, Dunn’s pairwise comparison with Bonferroni adjustment was performed after the Kruskal-Wallis test to compare stress hormones levels in healthy individuals and patients infected with different dengue serotypes. This has also been added to the statistical analysis section of the manuscript, and the relevant table (Table 4) has been updated accordingly.

*Line 217-218*

Shapiro-Wilk test was used to analyze the data normality.

*Line 222 - 226*

In addition, when a statistical association was found, pairwise analysis was performed using Dunn’s post hoc test with Bonferroni adjustment. Spearman’s correlation was used to assess the associations between viral load, stress markers, and hematological parameters in dengue-infected patients, and Bonferroni-adjusted p-values are reported.

-Adjust for Multiple Comparisons:

The study conducts multiple comparisons (e.g., hematological parameters across serotypes). To reduce the risk of Type I errors, apply corrections such as the Bonferroni or False Discovery Rate (FDR) method for multiple tests, ensuring more reliable p- values.

Response: *Table 5, Page no.26*

Thank you for your comment. As mentioned in our previous response, when a significant statistical association was observed, Dunn’s pairwise comparison with Bonferroni adjustment was performed after the Kruskal-Wallis test. However, for the comparison of hematological markers among patients with different dengue serotypes, multiple comparisons were not performed because the Kruskal-Wallis test did not show a significant difference between healthy individuals and patients with different dengue serotypes (Table 5).

-Increase Control Group Size:

The control group (n=15) is small compared to the patient group (n=150), potentially limiting the power of comparisons (e.g., Mann-Whitney U tests). Increasing the control group size would improve the robustness of differences observed in stress hormone levels.

Response: *Discussion Section, Page no. 11, Line no: 361-367*

This study was conducted during a dengue outbreak under limited-resource conditions, which restricted our ability to recruit a larger number of healthy control participants. The study was designed as exploratory and hypothesis generating rather than confirmatory. We agree that the small control group is a limitation, and as the study has already been completed, increasing its size is not feasible. This limitation has now been explicitly acknowledged in the Discussion as “Our study has certain limitations. First, the control group size was relatively small (n = 15) compared to the patient group (n = 150). Controls were recruited from healthy individuals during the outbreak period, but resource constraints limited their number. As a result, the study may have been underpowered to detect smaller associations, although it provided adequate data for descriptive analyses. Therefore, the findings should be interpreted as exploratory. Future larger, multi-center studies with formal sample size calculations will be needed to confirm and extend these preliminary observations.”

-Incorporate Phase-Specific Analysis:

The absence of fever phase data is a noted limitation. Stratify analyses by disease phase (febrile, critical, or recovery) to explore variations in stress hormone levels, which could strengthen the interpretation of cortisol and epinephrine findings.

Response: *Discussion Section, Page no. 12, line 370 - 374*

We fully agree with the reviewer that phase-specific analyses (febrile, critical, recovery) could provide valuable insights into stress hormone dynamics, this was not feasible within the constraints of our dataset.

In revised manuscript updated as, “Second, detailed phase-specific fever data were unavailable, which restricted our ability to evaluate fluctuations in stress hormone levels across different stages of illness. While demographics, hematological indices, study location, and study period were reported, unmeasured confounders such as timing of sample collection, comorbidities, and concurrent medications may have influenced cortisol and adrenaline levels.”

-Report Phylogenetic Confidence Measures:

The phylogenetic analysis lacks details on node confidence (e.g., bootstrap values). Include bootstrap or posterior probability values for the maximum likelihood tree generated by W-IQ-TREE to validate conclusions about multiple DENV-1 introductions.

Response: *Results Section, Page no. 9, Line no. 284-292*

In the revised manuscript we employed the same optimized phylogenetic pipeline as described in our previous publication [14] and updated as “Maximum likelihood phylogenetic analysis of DENV-1 sequences was performed using IQ-TREE2 (ver. 2.0.7) with the best-fit evolutionary model selected by ModelTest-NG, following the optimized pipeline previously reported in our earlier study [14]. The best-fit model for phylogenetic analyses of DENV-1 samples was TIM2+F+R, with three distinct clusters calculated by the ModelFinder [23] tool implemented in W-IQ-TREE. Node confidence was assessed using 1,000 ultrafast bootstrap replicates, and the resulting tree (Supplementary Figure S1) suggested multiple introductions of DENV-1 into Pakistan. The samples from NIH, Islamabad, Pakistan, belong to distinct lineages, indicating multiple introductions from other countries rather than a single introduction in Pakistan.”

- Discuss Clinical Relevance of Weak Correlations:

The Spearman correlations showed weak coefficients (e.g., r=0.184 for cortisol, r=-0.228 for hemoglobin). Provide a deeper discussion on the clinical significance of these weak associations and assess whether the sample size was sufficient to detect stronger relationships.

Response: *Discussion Section, Page no. 11, Line no. 346-349*

The clinical relevance of significant correlations has been discussed and added to the discussion section.

“In our investigation, we discovered a correlation between the measured blood parameters and stress hormones and virus load. Hemoglobin levels showed statistically significant inverse relationship with viral load.”
