# Supplementary material for: Profiles of stress hormones in relation to DENV serotypes among dengue-positive patients
Source: PLoS One. 2026 Apr 24;21(4):e0344532. doi: 10.1371/journal.pone.0344532 (PMC13108801; doi:10.1371/journal.pone.0344532)
Supplement: Attachment — Submitted filename: Response_to_Reviewers_auresp_2.docx [file pone.0344532.s006.docx]

**Response Sheet**

We would like to sincerely thank the reviewers for their time, effort, and valuable comments in reviewing our work. Their constructive feedback has greatly helped us refine and improve the manuscript, making it more meaningful and useful for the scientific community. Please find below our detailed responses to the reviewers’ comments, addressed point by point.

**Reviewer No 04:**

**General concern from the reviewer:** The biggest limitation of this study is the lack of data on fever days. Dengue is a very dynamic disease, changing day by day and even hour by hour. Therefore, laboratory data is also very dynamic. For example, during the first 3 days of fever (the febrile phase), we do not expect thrombocytopenia, and leukopenia usually occurs. Hemoglobin levels are also indirectly related; what is more accurately observed is the dynamics of hematocrit. There is also no data related to disease severity (with warning signs or severe dengue, although my assumption is that if hospitalized, they are not patients without warning signs). Stress hormones are certainly highly related to the dynamics of dengue disease progression and its severity. This is also what might cause the results to not show significance. Although each serotype may have varying severity, it also depends on the course of the illness (course of day-fever).

**Author’s Response:** Availability of extended clinical profile was a major limitation of the study. Since the study was conducted in a tertiary care hospital and the patients were recruited from the outpatient’s clinic, a complete record of clinical profile including fever and hospitalizations could not be managed. And for similar reasons it could potentially result in the variability of the laboratory data which cannot be mapped with the fever profile.

We agree that the relevance of stress hormones is not significant and that would potentially be due to the variation in available confounding factors including stages of the disease (early onset v/s recovery stage). However, the study would serve as the generation of the pilot data and would potentially serve as refinement of the second stage of the project.

**Questions:**

**Reviewer’s Question Q1**. In what way exactly is there a direct logical connection from discussing the newly emerging serotypes (such as DENV-3), as cited in Mendoza-Cano et al. in 2025, to the following assertion about self-managed and unreported cases of dengue infection occurring in Pakistan? And precisely how does this issue of underreporting thwart research efforts into identifying host physiological and hormonal responses to newly emerging serotypes in Pakistan?

**Author’s Response to Q1:** Although our cohort was based in Karachi, the report by Mendoza-Cano et al. (2025) highlights that dengue serotype distributions are dynamic and that serotypes such as DENV-3 are re-emerging in different regions. Our study relied exclusively on blood samples collected at a local tertiary care hospital and included only laboratory blood reports, without access to extended clinical or community-level data. Consequently, dengue infections that do not lead to hospital presentation are not biologically captured in our dataset. This directly explains the very low representation of DENV-3 in our cohort (1 out of 150 dengue-positive cases), and therefore no serotype-specific hormonal correlation analysis was performed for DENV-3. Thus, although emerging serotypes may be circulating in the population, the hospital-restricted sampling framework limits the ability to investigate host physiological and stress-hormone responses to these newly circulating strains.

The following information has been added to the discussion section page number ***Page no.10, Line 314-324***

“Although our cohort was based in Karachi rather than Mexico, the report by Mendoza-Cano et al. (2025) highlights that dengue serotype distributions are dynamic and that serotypes such as DENV-3 are re-emerging in different regions [27]. Our study relied exclusively on blood samples collected at a tertiary care hospital and included only laboratory blood reports, without access to extended clinical data. Consequently, dengue infections that do not lead to hospital presentation are not biologically captured in our dataset. This directly explains the very low representation of DENV-3 in our cohort (0.7%) i.e. 1 out of 150 dengue-positive cases) and precludes statistically meaningful analysis of serotype-specific physiological and stress-hormone responses. Thus, while emerging serotypes may be circulating in the population, the hospital-restricted sampling framework limits our ability to investigate host hormonal responses to these newly circulating strains.”

**Reviewer’s Question 2**.

**Author’s Response to Q2.** The reported study does not revolve around the characterization of the pathogen else than the molecular profile in terms of serotype and genotype. Although it would be convincing to relate the molecular profile of the virus to the cortisol level, a direct inference is not possible as the cortisol level can be modulated by the range of factors including patient own stress profile.

**Reviewer’s Question Q3**.

In regards to it being observed that your sources encompass research involving HSV and E71 but not necessarily related to DENV, what exactly would be the biologic rationale or evidential source from literature supporting such a postulate particular to the function of adrenaline in DENV replication? In addenda to this question, what would be the strength of your assertion about "adrenaline not contributing" to DENV replication based exclusively on "very weak correlation" to viral load?

**Author’s Response to Q3.**

We agree that studies on HSV and enterovirus 71 cannot be directly extrapolated to DENV, as viral tropism, replication compartments, and host–virus interactions differ substantially. These studies were cited only to highlight that catecholamines can modulate viral replication in some viral systems, not as evidence of a similar mechanism in dengue.

Furthermore, we acknowledge that the non-significant correlation observed between viral load and adrenaline levels in our cohort does not justify a definitive conclusion that adrenaline does not contribute to DENV replication. We have therefore revised the text to remove this assertion and now state that our data do not provide evidence supporting a role for adrenaline in DENV replication, and that mechanistic studies are required to address this question.

The following information has been added to the discussion section page number ***Page no. 11, Line 346-354***

“We also examined adrenaline, a stress hormone that has been scarcely studied in dengue infection. Our results showed lower epinephrine levels in dengue patients compared to controls, with relatively lower levels in those co-infected with DENV-1 and DENV-2. Previous studies in other viral systems, including enterovirus 71 and herpes simplex virus, have reported that catecholamines can modulate viral replication and infectivity [34, 35]; however, these findings cannot be directly extrapolated to dengue virus. In our cohort, only a very weak correlation was observed between viral load and adrenaline levels, which does not support a definitive role for adrenaline in DENV replication and highlights the need for targeted mechanistic studies in dengue models.”

**Reviewer’s Question Q4**.

Do patients co-infected with both DENV-1 and DENV-2 who had greater numbers of platelets have data related to other clinical features (such as severity grade, rate of hospitalizations due to severe dengue infection, ICU admissions rate, time to recovery) collected to provide direct supportive data to your hypothesis about "super-infection exclusion" contributing to reduced disease severity?

**Author’s Response to Q4.**

As mentioned above, the samples were collected from patients attending walk-in clinics, details relevant to ICU admissions or hospitalization could not be related.

**Reviewer’s Question Q5**.

Although it would be difficult to generalize to other regions internationally, what specific data suggests "the results do not appear to be confined to any particular sector or subgroup" in Karachi despite being carried out in a single tertiary-level hospital environment in the midst of a major outbreak?

**Author’s Response to Q5.**

Although the samples have been collected from a tertiary care hospital in Karachi, the data can be generalized as Karachi is one of the largest metropolitan sector of the country and caters a mass population of different background, multiple ethnicities and socioeconomic groups.
